# A novel dammarane triterpenoid alleviates atherosclerosis by activating the LXRα pathway

**DOI:** 10.1186/s13020-023-00758-0

**Published:** 2023-06-15

**Authors:** Yan Huang, Xiaodong Ran, Hongmei Liu, Mingming Luo, Yiyu Qin, Jinqiong Yan, Xiaohui Li, Yi Jia

**Affiliations:** Institute of Materia Medica and Department of Pharmaceutics, College of Pharmacy, Third Military Medical University, Shapingba, Chongqing, 400038 China

**Keywords:** Antiatherosclerosis, Ginsenoside compound K, Derivative, ATP-binding cassette A1, LXRα, Nuclear translocation

## Abstract

**Background:**

We have previously demonstrated that ginsenoside compound K can attenuate the formation of atherosclerotic lesions. Therefore, ginsenoside compound K has potential for atherosclerosis therapy. How to improve the druggability and enhance the antiatherosclerotic activity of ginsenoside compound K are the core problems in the prevention and treatment of atherosclerosis. CKN is a ginsenoside compound K derivative that was previously reported to have excellent antiatherosclerotic activity in vitro, and we have applied for international patents for it.

**Methods:**

Male C57BL/6 ApoE^−/−^ mice were fed a high-fat and high-choline diet to induce atherosclerosis and were subjected to in vivo studies. In vitro, the CCK-8 method was applied to evaluate cytotoxicity in macrophages. Foam cells were utilized, and cellular lipid determination was performed for in vitro studies. The area of atherosclerotic plaque and fatty infiltration of the liver were measured by image analysis. Serum lipid and liver function were determined by a seralyzer. Immunofluorescence and western blot analysis were conducted to explore the alterations in the expression levels of lipid efflux-related proteins. Molecular docking, reporter gene experiments and cellular thermal shift assays were used to verify the interaction between CKN and LXRα.

**Results:**

After confirming the therapeutic effects of CKN, molecular docking, reporter gene experiments and cellular thermal shift assays were used to predict and investigate the antiatherosclerotic mechanisms of CKN. CKN exhibited the greatest potency, with a 60.9% and 48.1% reduction in en face atherosclerotic lesions on the thoracic aorta and brachiocephalic trunk, reduced plasma lipid levels and decreased foam cell levels in the vascular plaque content in HHD-fed ApoE^−/−^ mice. Moreover, CKN in the present study may exert its antiatherosclerotic effects through activated ABCA1 by promoting LXRα nuclear translocation and reducing the adverse effects of LXRα activation.

**Conclusions:**

Our results revealed that CKN prevented the formation of atherosclerosis in ApoE^−/−^ mice by activating the LXRα pathway.

**Supplementary Information:**

The online version contains supplementary material available at 10.1186/s13020-023-00758-0.

## Introduction

Atherosclerosis is a pathological process that triggers life-threatening coronary artery disease and contributes for nearly 20% deaths worldwide [1]. Although several effective therapies are widely used to combat harmful lipids, serious cardiovascular diseases induced by atherosclerosis remain the major cause of health loss [2]. Therefore, the development of more effective therapeutic approaches based on new targets is of great importance. It is well acknowledgement that macrophages take up trapped modified and native LDL or lipoprotein remnants and differentiate into foam cells, which play a critical role in lesion plaque formation, and then can lead to atherosclerotic plaque disruption [3, 4]. The formation of foam cell involves an imbalance among cholesterol uptake, intracellular metabolism, and efflux [5, 6]. The modulation of cholesterol efflux has been suggested a potential relevant anti- atherosclerotic. Previous studies have found that the major transporter responsible for cholesterol efflux in macrophages is ATP-binding cassette transporter A1 (ABCA1) [7, 8], which regulates efflux to lipid-free apoA-I and is critical for high-density lipoprotein (HDL) particle biogenesis. The expression of ABCA1 is directly regulated by LXRα. The increased cellular cholesterol efflux caused by ABCA1seems to be a promising strategy to lower cardiovascular risk [9, 10]. GW3965 and T0901317, are the first two widely studied LXR agonists that induce the expression of target genes of both LXRα and LXRβ in vitro and in vivo. Although GW3965 and T0901317 are extensively used in experiment research as LXR agonists, they have proven unsuitable for clinical trials because of undesirable adverse effects, including hepatic steatosis and hypertriglyceridaemia [14]. Hence, it is necessary to explore new type LXR agonists which own acceptable lower adverse reactions.

*Panax notogin*seng (Burkill) F. H. Chen ex C. H. has been used as a natural remedy for cardiovascular protection in traditional Asian medicine for more than one thousand years [15]. Researches indicated that panax notoginseng saponins (PNS) are the major active components of *panax notogin*seng [16], which exhibit remarkable protective effects on atherosclerosis [17]. Ginsenoside compound K (CK, 20-O-d-glucopyranosyl-20(S)-protopanaxadiol) is one of the active metabolites (dammarane-typetriterpene) of panax notoginseng saponins [18]. Previous studies have demonstrated that CK has multiple pharmaceutical properties, including anti-osteoarthritis [19, 20], anti-tumour [21, 22], anti-diabetes [23, 24], anti-inflammatory [25, 26] and skin protective abilities [27]. our previous study has demonstrated that CK could attenuate the formation of atherosclerotic lesions in ApoE^−/−^ mice [28].

To improve the druggability and provide multiple hydrogen bond binding sites to improve the anti-atherosclerotic activity of Ginsenoside compound K, we previously demonstrated that CK hydroxyl protection products can significantly decrease cellular cholesterylester deposition in foam cells [29]. The results of this study indicated that these protective effects might be associated with the activation of ABCA1 [30]. The predominant pathway of CK metabolism involves the oxidation and cyclization of the hydroxyl and 24, 25-double bond. In addition, we achieved the structural modification of the carbon‒carbon double bond in CK on the basis of hydroxyl modification, among which CKN (Additional file 1: Scheme S2) showed excellent inhibition of macrophage foam cell accumulation [31]. We subsequently examined the effects of different doses of CKN on atherosclerotic plaques using high-fat fed ApoE^−/−^ mice, and found that CKN had better anti-atherosclerotic activity than atorvastatin and CK at an administered dose of 3 mg/kg (Additional file 1: Fig. S2). The chemical structure of CKN and related content of anti-atherosclerotic effects has formed an international patent [31].

In this article, we first examined the anti-atherosclerotic effects of CKN on ApoE^−/−^ mice. Then, we explored the possible role of LXRα-ABCA1 pathway activation in the pharmacological mechanism of CKN.

## Materials and methods

### General

Dulbecco’s Modified Eagle Medium (DMEM), 1640 Medium and fetal bovine serum were obtained from Hyclone (Invitrogen Corporation, NY, USA), CCK-8 reagents were obtained from Dojindo (Kumamoto, Japan), oxidized low density lipoprotein (ox-LDL, MDA 30 μM) was obtained from Institute of Basic Medicine, Peking Union Medical College (Beijing, China), anti-LXRα antibody, anti-ABCA1 antibody were obtained from novusbio Biologicals (IL, USA), anti-CD68 antibody was obtained from Sigma-aldrich (CA, USA), anti-IL-1β antibody and anti-TNF-α antibody were obtained from Santa Cruz Biotechnology (TX, USA), PE-conjugated secondary antibodies and PE-conjugated secondary antibodies were obtained from Biolegend Inc (SD, USA),Oil Red O, atorvastatin, T-PER protein extraction reagent and SR9243 were obtained from Sigma-Aldrich (CA, USA), reporter genes were synthesized by Shanghai Genechem Co.,Ltd. (Shanghai, China), IL-1β and TNF-α ELISA kit were obtained from Dakewe Biotechnology (Beijing, China),BCA assay kit was obtained from Beyotime (Shanghai, China), bovine serum albumin was obtained from Sangon Biotech (Shanghai, China), HRP-conjugated anti-β-actin antibody was obtained from Kangchen Inc. (Shanghai, China).

### Animals and treatments

To study the preventive and therapeutic effects of compound on atherosclerosis in vivo, 72 male C57BL/6 ApoE^−/−^ mice aged 8 weeks (22–25 g) were purchased from Peking University Health Science Center (Beijing, China). The mice were housed under specific pathogen-free conditions on a 12-h light–dark cycle in the animal facility at the Animal Center of the Third Military Medical University. Animals were randomly divided into five groups (n = 6) and provided with unlimited access to water and high-fat and high-choline diet (basic feed containing 40% fat and 1.25% cholesterol and 0.5% sodium cholate), except the control group with basic feed. Animals were treated with CKN (3 mg/kg), CK (3 mg/kg) and atorvastatin (3 mg/kg) by intra-peritoneal injection once a day. After 10 weeks, the mice were fasted overnight and sacrificed by CO_2_ inhalation in accordance with the AVMA guidelines for the euthanasia of animals, 2013 edition. Samples were collected for the subsequent experiments.

### Histology and image analysis of aortic

For en face analysis, the thoracic aorta and brachiocephalic trunk were opened longitudinally and fixed by 10% formalin. The samples were stained with Oil-Red O for 15 min and washed. The cross-sections of aorta root were separately stained with hematoxylin/eosin (H&E), and cross-sections of aorta root were separately stained with CD68 and ABCA1. We used 4% PFA solution to fix mouse liver tissues. Sections of the liver samples or the frozen liver tissues were stained with H&E or Oil Red O. The sections were examined under a light microscope Images were captured with Laser Confocal Microscope (Nikon Eclipse 90i light microscope), Image-Pro Plus 6.0 software were used to aortic tissues semiquantitative analysis.

### Serum lipid and cytokines

Blood sample was collected from the abdominal vena cava and the plasma supernatants were collected for further study. The levels of total cholesterol (TC), low-density lipoprotein cholesterol (LDL-C), triglycerides (TG), and high-density lipoprotein cholesterol (HDL-C) were detected using an AU-2700 automatic biochemical analyzer (Olympus, Tokyo, Japan). The serum cytokine levels, including tumor necrosis factor-α (TNF-α) and interleukin-1β (IL-1β), were detected using a Mcytomag-70 K-3 Mouse Cytokine/Chemokine Magnetic Bead Panel (Merck Millipore Co. LTD).

### Immunofluorescence

The murine macrophage cell line RAW264.7 was obtained from the Cell Bank of the Shanghai Institutes for Biological Sciences, Chinese Academy of Sciences. Immunofluorescence assay was performed to detect the expression and the distribution of CD68 and ABCA1 in the aortic tissues, or LXRα and ABCA1 in RAW 264.7 macrophages. After being blocked by goat serum for 60 min at room temperature, the samples were incubated with primary antibodies overnight at 4 °C. Subsequently, the samples were treated with the corresponding PE-conjugated secondary antibodies or FITC-conjugated secondary antibodies for 60 min at room temperature and 4, 6-diamidino-2-phenylindole (DAPI) in the dark. Images were visualized under a fluorescence microscope (OLYMPUS fv1000, Japan) at 400 × magnification.

### Cytotoxicity assay

Cellular toxicity assays were carried out using the CCK-8 method. RAW 264.7 macrophages were seeded in 96-well plates at 5 × 10^4^/mL in RPMI-1640 containing 10% fetal bovine serum. The cells were treated with 3, 10, or 30 μM of CKN for 24 h. Ten microliters per well of CCK-8 reagent was then added. The cells were incubated at 37 °C for 1 h, and the OD value was measured at 450 nm by SpectraMax M3 microplate reader (Molecular Devices, CA, USA). The experiments were performed in triplicate.

### Intracellular ox-LDL internalization stained by oil red O

RAW264.7 cells were cultured in 6-well plates (5 × 10^5^/mL), cells were processed as follows: (a) the control group was treated with 1640 culture medium, (b) the model group was treated with 100 μg/ml ox-LDL for 24 h, (c) the DMSO group was treated with 100 μg/ml ox-LDL + 0.5%DMSO for 24 h, (d) the CKN group was treated with different concentrations of CKN dissolved in DMSO along with 100 μg/mL ox-LDL for 24 h. Then the cells fixed with 4% paraformaldehyde and then stained with Oil Red O, red stained intracellular internalization ox-LDL was visualized under a phase-contrast microscope (Leica DM500). Subsequently, washed Oil Red O-stained cellular lipid was extracted by isopropanol (250 μL/well), and the optical density at 500 nm was determined. The OD values were calculated relative to the model group. The experiments were performed in quintuplicate.

### In vitro anti-inflammatory effect

The anti-inflammation effect of CKN on macrophages was conducted in 24-well plates. Cells were incubated with 100 ug/mL ox-LDL, meanwhile treated by CKN for 24 h. Then, culture media were harvested to detect expression of tumor necrosis factor-α (TNF-α) and interleukin-1β (IL-1β) by ELISA kit.

### Western blotting

After treatment, aorta tissue or cells were homogenized on ice for 10–20 min in 50 mM T-PER, 150 mM NaCl, and 2 mM DTT (Thermo Fisher Scientific), and total protein concentrations were determined using a bicinchoninic acid protein assay kit (Thermo Fisher Scientific). Equal amounts of protein extracts were separated using 10% sodium dodecyl sulphate polyacrylamide gel electrophoresis. After blocking by 5% defatted milk for 1 h at room temperature, the PVDF (Millipore, USA). membrane with protein on it was treated with the primary antibodies overnight at 4 °C and corresponding secondary antibody for 30 min at room temperature. After washing with TBST, membranes were incubated with a horseradish peroxidase-conjugated secondary antibody for 1 h at room temperature. Finally, proteins were visualized using an enhanced chemical luminescent system (BLT GelView 6000 Pro).

### Dock into the LXRα

Chem Draw 12.0 program was used for converting the chemical structures into 3D conformation. Ligand preparation was done using Pymol 1.8.6. The crystal structure of mouse LXRα (PDB entry: 5 AVL) was prepared by SYBYL-X 2.0. After the software validation by re-docking method with respective reference ligands CKN was docked into the active site of the LXRα within the radius of 6.5 Å (default). Other parameters were set referring the default values. The docking scores and suitable binding patterns were reported by comparing with the reference ligands.

### Reporter gene experiment

HEK293T cells were adjusted to 5 × 10^5^/mL in DMEM containing 10% (v/v) fetal bovine serum. LXR-α-pcDNA3 or LXR-β-pcDNA3, LXRE-Luc (reporter plasmid), and pSV-β-galactosidase (transfection efficiency control) were transfected into HEK293T cells, and the cells were cultured for 48 h prior to further treatments. Different concentrations of CK and CKN were added into the medium and GW3965 (10 μM) was used as positive control.

### Cellular thermal shift assay

The RAW264.7 cells were collected and lysed in 50 mM T-PER, 150 mM NaCl, and 2 mM DTT. Then two groups divided in EP tubes. One group was mixed with 30 μM CKN, and the other was mixed with 0.5% DMSO as a negative control. The samples were heated at 45–69 °C under the same conditions using a heat block. Each sample was heated at a single temperature for 2 min, placed on ice, and centrifuged at 12,000 g for 30 min. The supernatant was collected for western blotting testing.

## Results

### CKN alleviates atherosclerosis in ApoE^−/−^ mice

**Fig. 1:**
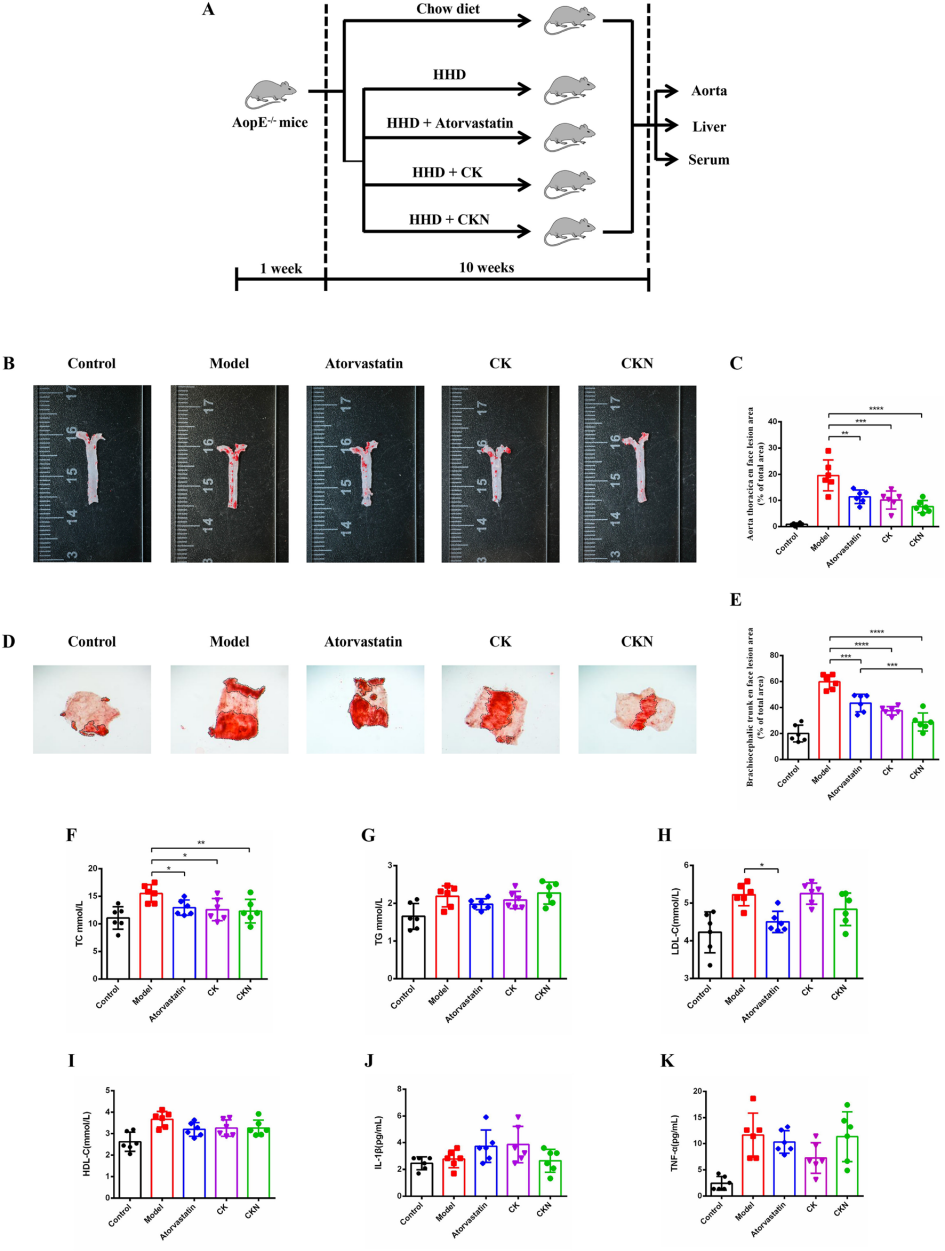
CKN attenuated the formation of atherosclerotic lesions in ApoE^−/−^ mice. The design of the experiments (**A**). Representative images of aortic lesions in the thoracic aorta (**B**) and quantification of atherosclerotic lesions shown as the percentage of thoracic aorta (**C**). Representative images of aortic lesions in the brachiocephalic trunk (**D**) and quantification of atherosclerotic lesions shown as a percentage of the brachiocephalic trunk (**E**). The serum lipid levels of total cholesterol (TC) (**F**), total triglyceride (TG) (**G**), low-density lipoprotein-cholesterol (LDL-C) (**H**) and high-density lipoprotein-cholesterol (HDL-C) (**I**). Serum and local levels of IL-1β (**J**) and TNF-α (**K**) were modulated by CKN. Data are presented as mean ± SD (n = 6) and analyzed by ANOVA with Dunnett’s post-hoc analysis. *P < 0.05, **P < 0.01; ***P < 0.001, ****P < 0.0001

To investigate the effect of CKN on the progression of atherosclerosis in vivo, a clinical dose of 3 mg/kg was administered once daily to ApoE^−/−^ mice after 10 weeks of high-fat diet feeding (Fig. 1A). Compared with the model group, atorvastatin, CK and CKN significantly reduced the atherosclerotic lesions in the thoracic aorta and brachiocephalic trunk (Fig. 1B–E), where the inhibition rates of Atorvastatin, CK and CKN on the thoracic aorta plaque were 42%, 48% and 61%, respectively. And the inhibition rates of the brachiocephalic trunk plaque were 27%, 37% and 52%, respectively. CKN has a significant advantage in inhibiting plaque formation in thoracic aorta and brachiocephalic trunk compared with Atorvastatin, CK and CKN significantly reduced the level of total cholesterol (TC), as compared to Atorvastatin and CK were not significantly different. However, the levels of triglycerides (TG), low-density lipoprotein cholesterol (LDL-C) and high-density lipoprotein cholesterol (HDL-C) did not change significantly compared to the model group (Fig. 1F–I). Compared to the model group, the CKN treatment group exhibited decreased serum IL-1β levels, and superior to Atorvastatin and CK (Fig. 1J).

**Fig. 2:**
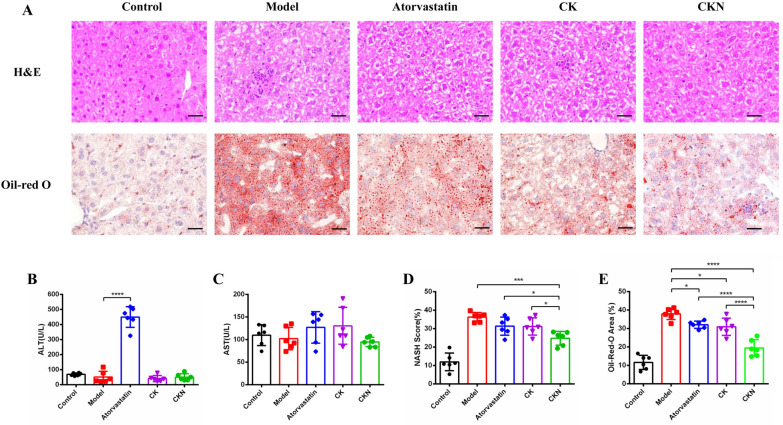
CKN did not cause hepatotoxicity. Representative images of H&E- and Oil Red O-stained (**A**) liver sections. Scale bars: 500 µm. Detection of ALT (**B**) and AST (**C**) in ApoE^−/−^ mice after different treatments. Changes in the NASH SCORE in each group (**D**). Quantification of the lipid content in the liver with Oil Red O staining (n = 6) (**E**). Data are presented as mean ± SD (n = 6) and analyzed by ANOVA with Dunnett’s post-hoc analysis. *P < 0.05, ***P < 0.001, ****P < 0.0001

**Fig. 3:**
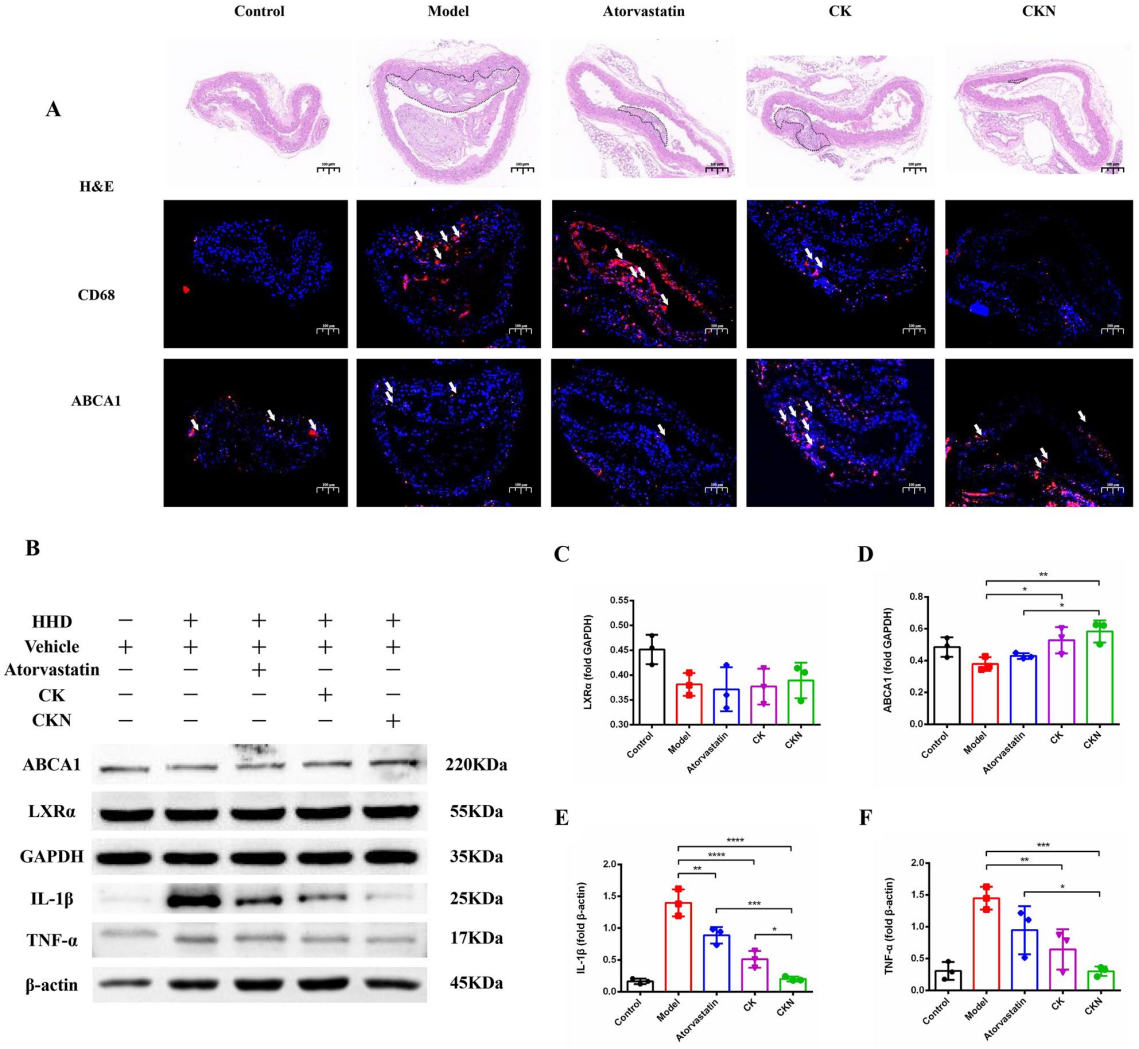
CKN reduced the inflammatory reaction in the aorta. Representative histochemistry images of aortic root cross-sections stained with CD68 antibody and ABCA1 antibody showed that CKN treatment decreased macrophage infiltration in the aorta. Scale bars: 100 µm (**A**). The levels of LXR signaling pathway and inflammatory cytokine proteins in atherosclerotic mice were determined using Western blotting (**B**). Western blot analysis of LXRα (**C**) and ABCA1 (**D**) in aortic tissue treated with CKN. The western blotting results showed that CKN treatment significantly decreased the levels of IL-1β (**E**) and TNF-α (**F**) in aortic tissue. Data are presented as mean ± SD (n = 3) and analyzed by ANOVA with Dunnett’s post-hoc analysis. *P < 0.05, **P < 0.01; ***P < 0.001, ****P < 0.0001

At the same time, we adopted CD68 immunofluorescence to detect the anti-inflammatory effect of CKN on plaques. As expected, the level of CD68 (a marker for macrophage infiltration) was significantly reduced after CKN treatment, As expected, aortic root CD68 (a marker of macrophage infiltration) levels were significantly reduced after CK and CKN treatment, whereas Atorvastatin had no effect on aortic root CD68 expression, and CKN had the best effect (Fig. 2A). Cholesterol efflux is an important factor affecting macrophage foaming, and LXRα and ABCA1 are major transporters responsible for cholesterol efflux. Therefore, we detected changes in ABCA1 expression in aortic root plaques after Atorvastatin, CK and CKN treatment. Aortic immunofluorescence and Western blot analyses revealed that CKN significantly increased ABCA1 expression compared to Atorvastatin and CK. (Fig. 2A and D). Nevertheless, western blotting analysis revealed that CKN did not increase the expression of LXRα (Fig. 2B and C). Furthermore, CKN down regulated IL-1β and TNF-α level in vascular tissues more significantly than Atorvastatin and CK (Fig. 2E and F). Judging by the liver function tests, CKN treatment did not cause significant hepatotoxicity (Fig. 3B and C). Histological analysis revealed that CKN reduced lipid deposition and hepatic damage caused by HHD. However, both Atorvastatin and CK groups had severe hepatic lipid deposition (Fig. 3A, D and E). These findings demonstrate that CKN can effectively prevent the development of atherosclerosis in ApoE^−/−^ mice by up-regulating ABCA1 expression in macrophages. And CKN do not cause hepatotoxicity in mice and also attenuate hepatic lipid deposition.

### CKN inhibits macrophage foam cell accumulation and the deposition of cholesteryl esters in foam cells

**Fig. 4:**
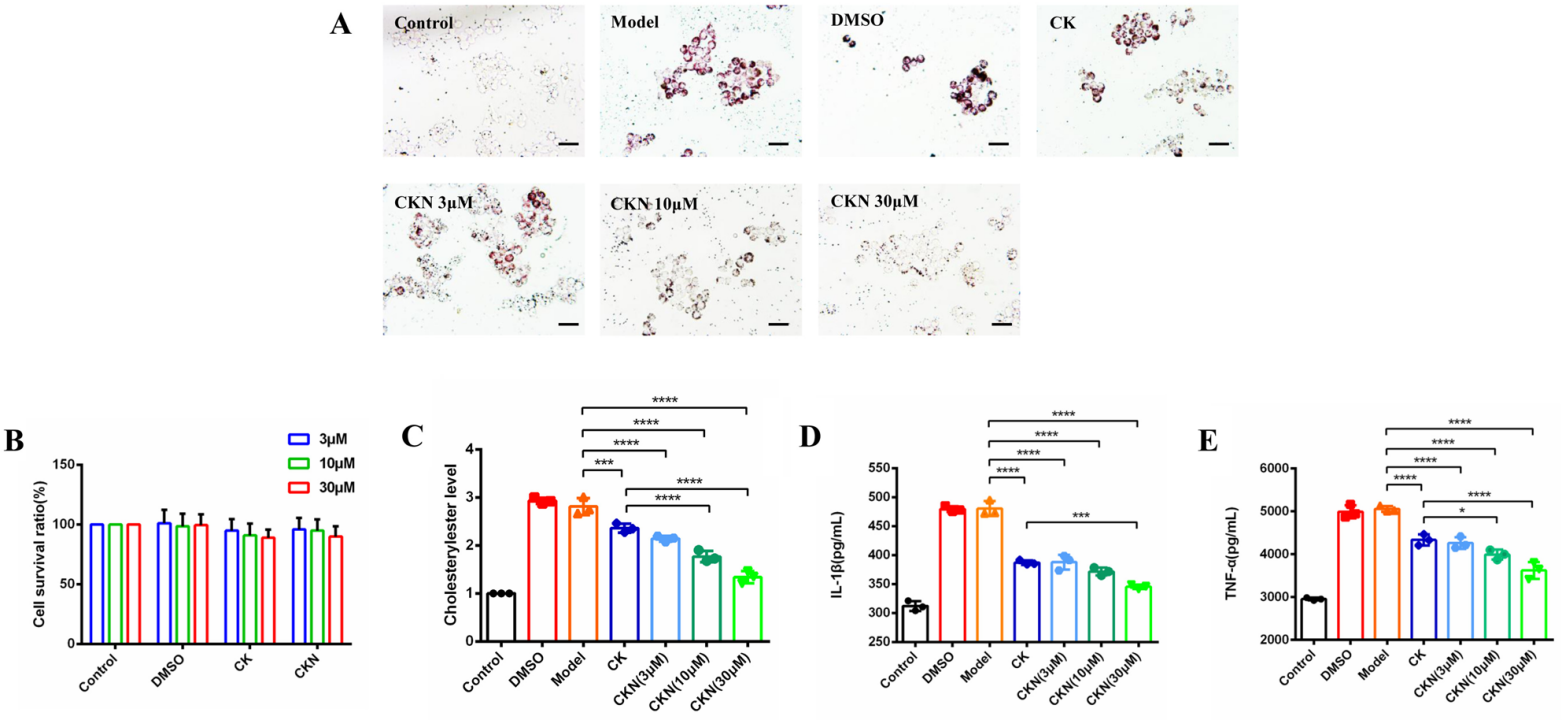
CKN inhibited the formation of foam cells. The concentrations of cholesteryl ester in foam cells decreased after RAW264.7 macrophages were treated with CK (30 μM) or CKN (3, 10, and 30 μM). Scale bars: 50 µm (**A** and **C**). The cellular toxicity of CKN was analysed using the CCK-8 method (**B**). Cellular IL-1β (**D**) and TNF-α (**E**) levels decreased after treatment with CKN (3, 10, and 30 μM). Data are presented as mean ± SD (n = 3), and significance was determined by ANOVA with Dunnett’s post-hoc analysis. *P < 0.05, **P < 0.01; ***P < 0.001, ****P < 0.0001

To verify the cytotoxicity of CKN towards RAW264.7 macrophages, we assessed cell viability using the CCK-8 assay. CKN showed no significant cytotoxicity to foam cells at different dosages compared to the control group (Fig. 4B). Hence, 3, 10 and 30 μM CKN were identified as safe dosages for in vitro assays. Compared to the model and CK group, CKN effectively decreased the deposition of cholesteryl esters in RAW264.7 macrophage foam cells at concentrations of 10 μM and 30 μM (Fig. 4A and C). We found that the levels of TNF-α and IL-1β after ox-LDL treatment were higher in RAW264.7 macrophage cells than in controls, while ckn decreased the levels of TNF and IL more effectively than the same concentration of CK. (Fig. 4D, E).

### CKN actives LXRα and increases ABCA1 expression

**Fig. 5:**
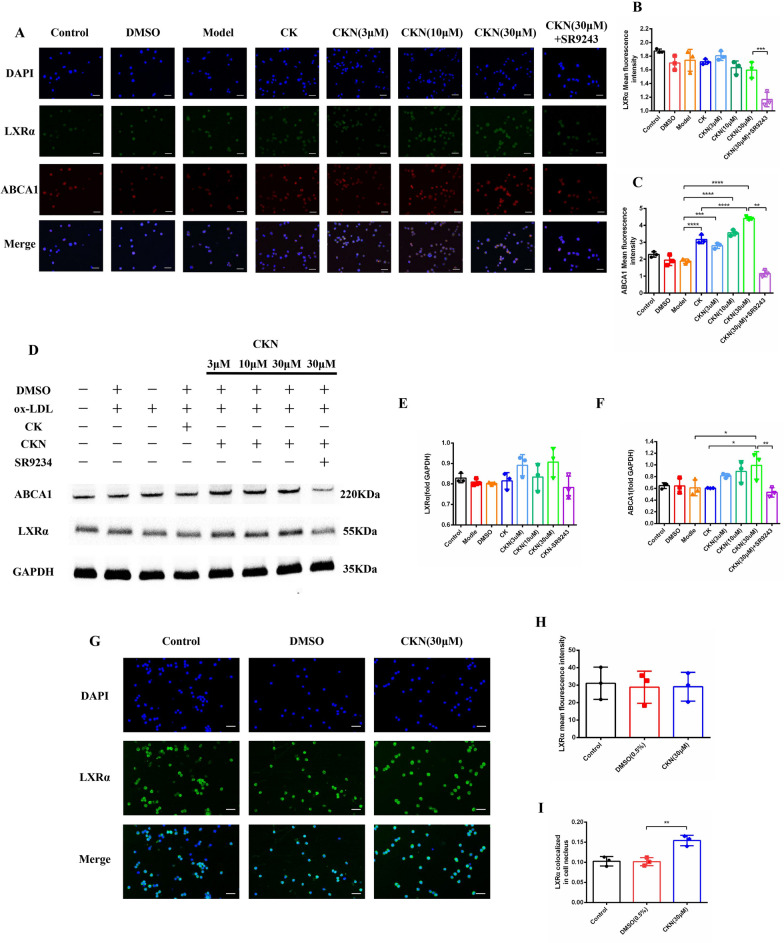
The pharmacological effects of CKN may be associated with the LXRα pathway. RAW264.7 macrophages were stimulated with 50 µg/mL ox-LDL, 50 µg/mL ox-LDL + CKN (30 μM) and 50 µg/mL ox-LDL + CKN (3, 10, and 30 μM) for 24 h. The cells were fixed with 4% paraformaldehyde and stained with DIPY (blue fluorescence), anti-LXRα antibody (green fluorescence) and anti-ABCA1 antibody (red fluorescence). Scale bars: 50 µm (**A**). Quantitative analysis of LXRα and ABCA1 fluorescence intensity (**B** and **C**). Western blotting was used to examine LXRα and ABCA1 protein expression in CKN-treated or untreated macrophages (**D**). Western blot analysis of LXRα (**E**) and ABCA1 (**F**) in macrophages treated with CKN. RAW264.7 macrophages were treated with CKN (100 μM) for 24 h. LXRα (green fluorescence) was found in the cytoplasm of RAW264.7 cells. Scale bars: 50 µm (**G**). CKN treatment did not change the total expression of LXRα (**H**). After CKN treatment, the LXRα (green fluorescence) signal was significantly decreased in the cytoplasm but increased in the nucleus (blue fluorescence) (**I**). Data are presented as mean ± SD (n = 3) and were analyzed by ANOVA with Dunnett’s post-hoc analysis. *P < 0.05, **P < 0.01; ***P < 0.001

The animal experiment results suggested that CKN can effectively prevent the development of atherosclerosis in ApoE^−/−^ mice by upregulating ABCA1 expression in macrophages. Therefore, we discussed the mechanism of action of CKN through RAW264.7 cell experiments. ABCA1 is a major downstream gene of the LXRα pathway [14]. The results showed that treatment with CKN significantly increased the levels of ABCA1 at the same time compared with the model and CK groups. Treatment with the LXRα antagonist SR9243 reversed the alteration in ABCA1 expression (Fig. 5A–F).

**Fig. 6:**
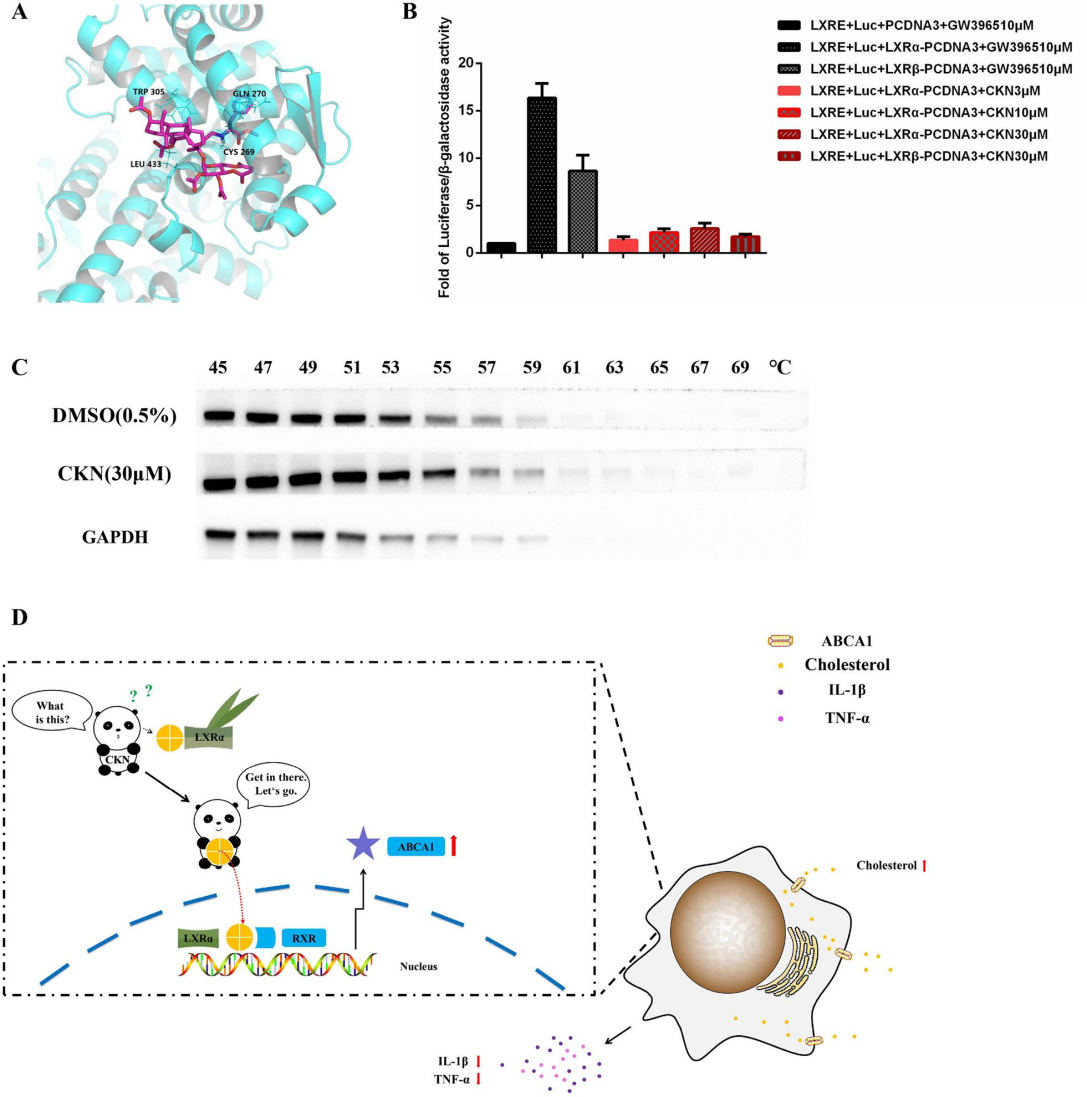
CKN is a novel LXRα agonist. Docking of CKN to mouse LXRα was simulated by SYBYL-X 2.0 (**A**). HEK293T cells were treated with different concentrations of CKN, and GW3965 (10 μM) was used as a positive control (**B**). The RAW264.7 cell proteins were mixed with compound CKN (30 μM), and the control group was treated with 0.5% DMSO. The proteins were heated at 45 to 69 °C under the same conditions using a heat block for 2 min. The supernatant was collected for western blotting (**C**). Diagram of the molecular mechanisms of CKN (**D**). Data are presented as mean ± SD (n = 3) and were analyzed by ANOVA with Dunnett’s post-hoc analysis

To explore the effect of CKN on LXRα, we performed rigid docking (Fig. 6A) and CETSA [32] (Fig. 6C) studies of CKN. The results suggested that CKN can bind to LXRα. Furthermore, we measured the effects of LXRα activation with a gene reporter method. The results suggested that CKN has minimal effects on the expression of LXRα (Fig. 6B). Furthermore, we used 30 μmol/L as the optimal dose in immunofluorescence experiments. As shown in the immunofluorescence results, the expression levels of LXRα in RAW264.7 cells were unchanged after treatment with CKN, but CKN upregulated the expression of LXRα in the nucleus compared to that in the DMSO group (Fig. 5G–I).

## Discussion

Cardiovascular disease (CVD) comprises a wide range of disorders, including myocardial infarction and stroke, for which atherosclerosis is the major pathological mechanism. CVD has surpassed infectious diseases and become the main cause of death and disability worldwide [33]. It is widely believed that atherosclerosis is a chronic inflammatory disease induced by risk factors such as hyperlipidaemia and hypertension [34, 35]. At present, the clinical prevention and treatment modalities include using HMG-CoA reductase inhibitor statins to reduce plasma LDL-C, but the incidence of serious cardiovascular and cerebrovascular diseases related to atherosclerosis is still on the rise in the context of widely used statins to achieve the expected lipid regulation level [36].

To evaluate the therapeutic effect of CKN, its anti-atherosclerotic abilities were assessed. Based on the results of our in vitro experiments, we explored the anti-atherosclerotic effects of CKN in the ApoE^−/−^ mouse model. CKN exhibited greater potency than atorvastatin in decreasing the area of en face atherosclerotic lesions and decreasing the foam cell level of vascular plaques. Furthermore, derivative treatment resulted in decreased TC in serum and reduced inflammatory cytokine levels, including a decrease in pro-inflammatory cytokine levels in the aorta. Nevertheless, the molecular mechanisms through which CKN regulates atherosclerotic lesion metabolism remain unclear.

Animal experiments thus suggested that CKN exerts beneficial anti-atherosclerotic effects by modulating the expression of the ABCA1 gene. Clinical studies have shown that carriers of ABCA1 functional loss mutations are characterized by reduced levels of high-density lipoprotein cholesterol, which is found in tissues rich in macrophages and reticular endothelial cells, leading to atherosclerosis [37]. ABCA1 plays a central role in the mechanism of cholesterol efflux from macrophages, mediating reverse cholesterol transport and preventing harmful lipid deposition. ABCA1 deficiency in macrophages has been reported to increase inflammation and accelerate atherosclerosis in mice [38, 39].

To explore the possible mechanism of the up-regulation of ABCA1, we detected the expression of LXRα. As reported previously, ABCA1 is a downstream gene of LXRα; therefore, the activation of LXRα will increase the expression of ABCA1 [14], which is considered to have anti-inflammatory effects and to modulate serum lipid levels [40]. Our results showed that CKN treatment did not alter the expression of LXRα in cells but significantly increased the nuclear translocation of LXRα. Since nuclear translocation is a necessary step in the implementation of the biological effect of LXRα, the increase in nuclear translocation could indicate the activation of LXRα. Together with the results regarding the unchanged levels of total LXRα protein expression and the reporter gene experiments, it is suggested that the pharmacological effects of CKN were induced by direct binding to LXRα but not the upstream target. In addition, CKN activated ABCA1 by promoting LXRα nuclear translocation without undesirable adverse effects such as liver fat deformation or hypertriglyceridaemia.

In summary, a novel synthesized dammarane triterpenoid, CKN, presented remarkable preventive effects against the formation of atherosclerosis in ApoE^−/−^ mice in this study. The mechanism is associated with dual-action in cholesterol metabolism and the inflammation reaction by activating the LXRα pathway. It also reduces the adverse effects of LXRα activation.

## Supplementary Information

**Additional file 1: Scheme S1.** Syntheses of Compound K derivatives 1-3.Ac_2_O−C_5_H_5_N, 60°C, 24h, 90%;mCPBA,CH_2_Cl_2_, rt , 4h, 95%;HIO_4_, CH_3_CN−H_2_O, 0^O^C, 3h, 85%. **Scheme S2.** Syntheses of CKN. Reagents and conditions: RNH_2_, NaBH_3_, DCM, rt. **Figure S1.** The NMR spectrum analysis of 1, 2, 3 and CKN. The ^1^H NMRand ^13^C NMRof 1, solvent: CDCl_3_. The ^1^H NMRof 2, solvent: CDCl_3_. The ^1^H NMRand ^13^C NMRof 3, solvent: CDCl_3_. The ^1^H NMRand ^13^C NMRof CKN, solvent: CDCl_3_. **Figure S2.** Effencts of different administration doses of CKN on atherosclerotic plaques in AopE^−/−^ mice. Representative images of thoracic aortic lesionsand quantification of atherosclerotic lesions shown as percentage of thoracic aorta.

## Data Availability

Not applicable.

## Abbreviations

CK: Ginsenoside compound K
LXRα: Liver X receptor α
ABCA1: ATP-binding cassette A1
ox-LDL: Oxidized low density lipoprotein
DMEM: Dulbecco’s Modified Eagle Medium
TC: Total cholesterol
LDL-C: Low-density lipoprotein cholesterol
TG: Triglycerides
HDL-C: High-density lipoprotein cholesterol
ECL: Enhanced chemiluminescence
BSA: Bovine serum albumin
TNF-α: Tumor necrosis factor-α
IL-1β: Interleukin-1β
DAPI: 4′, 6-Diamidino-2-phenylindole
DMSO: Dimethyl sulfoxide
