# Supplementary material for: A novel dammarane triterpenoid alleviates atherosclerosis by activating the LXRα pathway
Source: Chin Med. 2023 Jun 15;18:72. doi: 10.1186/s13020-023-00758-0 (PMC10273626; doi:10.1186/s13020-023-00758-0)
Supplement: Supplementary file 1 — Additional file 1: Scheme S1. Syntheses of Compound K derivatives 1-3.Ac2O−C5H5N, 60°C, 24h, 90%;mCPBA,CH2Cl2, rt , 4h, 95%;HIO4, CH3CN−H2O, 0OC, 3h, 85%. Scheme S2. Syntheses of CKN. Reagents and conditions: RNH2, NaBH3, DCM, rt. Figure S1. The NMR spectrum analysis of 1, 2, 3 and CKN. The 1H NMRand 13C NMRof 1, solvent: CDCl3. The 1H NMRof 2, solvent: CDCl3. The 1H NMRand 13C NMRof 3, solvent: CDCl3. The 1H NMRand 13C NMRof CKN, solvent: CDCl3. Figure S2. Effencts of different administration doses of CKN on atherosclerotic plaques in AopE−/− mice. Representative images of thoracic aortic lesionsand quantification of atherosclerotic lesions shown as percentage of thoracic aorta. [file 13020_2023_758_MOESM1_ESM.doc]

**Supplementary** **material**

**Synthesis and characterization of Ginsenoside CKN**

Scheme S1. Syntheses of Compound K derivatives 1-3. (a) Ac_2_O−C_5_H_5_N, 60°C, 24h, 90%; (b) mCPBA,CH_2_Cl_2_, rt , 4h, 95%; (c) HIO_4_, CH_3_CN−H_2_O, 0^O^C, 3h, 85%;

Scheme S2. Syntheses of CKN. Reagents and conditions: RNH_2_, NaBH(Ac)_3_, DCM, rt.

**Materials and methods**

**General**

All the starting materials were of reagent grade. The solvents used for the isolation/purification of the compounds were obtained from J&K Scientific LTD (Beijing, China). All reactions were carried out in oven-dried glassware under an argon atmosphere unless otherwise noted. All yields reported refer to the yields of the isolated compounds. ^1^H NMR and ^13^C NMR spectra were recorded using a Varian Inova-600 spectrometer (600 MHz). High-resolution mass spectra were obtained with an HRMS (Agilent-6120, Agilent) mass spectrometer. Silica gel TLC plates (Qing Dao Marine Chemical Factory, Qingdao, China) were used to monitor the progression of the reactions. Flash column chromatography was performed using silica gel (200−400 mesh size, Qing Dao Marine Chemical Factory, Qingdao, China).

**General procedure for the synthesis of 1.**

A solution of compound K (1 g, 1.61 mmol) in pyridine (10 mL) was mixed with Ac_2_O (10 mL), and the mixture was stirred at 60 °C for 24h. Then the mixture was added to a lot of water and the product was isolated by extraction with dichloromethane. The organic phase was washed with lye, brine, dried over anhydrous sodium sulfate, filtered, then Silica gel was added and concentrated under vacuum. After evaporation of excess reagent, the residue was subjected to column chromatography on silica gel using PE/EtOAc (5/1, V/V) to yield white powder (1.33g, 1.52mmol, 95%).

^1^H NMR (600 MHz, cdcl3) δ 5.17 (t, J = 9.5 Hz, 1H), 5.03 – 4.94 (m, 2H), 4.90 (t, J = 8.7 Hz, 1H), 4.81 (td, J = 10.6, 4.7 Hz, 1H), 4.65 (d, J = 7.8 Hz, 1H), 4.46 (dd, J = 11.0, 4.1 Hz, 1H), 4.13 – 4.05 (m, 2H), 3.66 – 3.60 (m, 1H), 2.03 (s, 3H), 2.03 (s, 3H), 2.01 (s, 3H), 2.00 (s, 3H), 1.97 (s, 3H), 1.96 (s, 3H).

^13^C NMR (151 MHz, cdcl3) δ 170.77, 170.58, 170.29, 170.17, 169.43, 168.98, 131.46, 124.45, 94.70, 83.19, 80.61, 75.12, 73.30, 71.95, 71.53, 68.90, 62.59, 55.88, 53.05, 49.92, 47.76, 45.56, 39.52, 39.02, 38.46, 37.84, 36.97, 34.42, 31.68, 29.09, 27.92, 26.36, 25.58, 23.53, 22.77, 21.84, 21.76, 21.20, 20.73, 20.59, 20.56, 20.55, 18.14, 18.01, 17.65, 16.40, 16.14, 15.39.

HRMS(ESI+) m/z calcd. for C48H74O14 [M + Na]+ 897.4976, found 897.5037.

**General procedure for the synthesis of 2** [1, 2]**.**

A solution of compound 1 (1 g, 1.14 mmol) in DCM (10 mL) was mixed with mCPBA (0.21 g, 1.2mmol), and the mixture was stirred at rt for 0.5h. Then The reaction mixture was partitioned with EtOAc and H_2_O. The organic phase was washed with lye, brine, dried over anhydrous sodium sulfate, filtered, then Silica gel was added and concentrated under vacuum. After evaporation of excess reagent, the residue was subjected to column chromatography on silica gel using PE/EtOAc (5/1, V/V) to yield white powder (0.91 g, 1.02μmol, 90%).

^1^H NMR (600 MHz, cdcl_3_) δ 5.17 (t, *J* = 9.5 Hz, 1H), 5.00 (t, *J* = 9.7 Hz, 1H), 4.92 – 4.84 (m, 1H), 4.86 – 4.75 (m, 1H), 4.68 (dd, *J* = 20.8, 7.9 Hz, 1H), 4.46 (dd, *J* = 11.2, 4.2 Hz, 1H), 4.13 (dd, *J* = 20.1, 3.6 Hz, 2H), 3.66 – 3.61 (m, 1H), 2.60 (ddd, *J* = 18.4, 7.6, 4.2 Hz, 1H), 2.15 – 1.95 (m, 18H).

HRMS(ESI+) m/z calcd. for C_48_H_74_O_15_[M + Na]+ 913.4925, found 913.4981.

**General procedure for the synthesis of 3** [3, 4]**.**

A solution of compound 1 (0.9 g, 1 mmol) in mixture of acetonitrile and water (AC: H_2_O=3:1, V/V, 10 mL) was mixed with HIO_4_ (0.24 g, 1.1mmol), and the mixture was stirred at 0°C for 2h. Then The reaction mixture was partitioned with EtOAc and H_2_O. The organic phase was washed with lye, brine, dried over anhydrous sodium sulfate, filtered, and then Silica gel was added and concentrated under vacuum. After evaporation of excess reagent, the residue was subjected to column chromatography on silica gel using PE/EtOAc (5/1, V/V) to yield white powder (0.73 g, 0.86mmol, 85%).

^1^H NMR (600 MHz, cdcl_3_) δ 9.74 (s, 1H), 5.16 (t, *J* = 9.5 Hz, 1H), 4.97 (t, *J* = 9.8 Hz, 1H), 4.92 – 4.87 (m, 1H), 4.80 (td, *J* = 10.6, 5.2 Hz, 1H), 4.69 (dd, *J* = 25.5, 14.5 Hz, 1H), 4.49 – 4.42 (m, 1H), 4.18 – 3.99 (m,2H), 3.66 (ddd, *J* = 13.2, 12.1, 6.2 Hz, 2H), 3.38 (dd, *J* = 14.1, 7.1 Hz, 1H), 3.30 – 3.22 (m, 1H), 2.75 – 2.65 (m, 1H), 2.39 – 2.28 (m, 1H), 2.16 – 2.08 (m, 1H), 2.08 – 1.94 (m, 18H).

^13^C NMR (151 MHz, cdcl_3_) δ 202.10, 170.84, 170.63, 170.39, 170.32, 169.41, 168.96, 94.59, 82.94, 80.52, 75.20, 73.08, 71.77, 71.61, 68.40, 62.07, 55.77, 52.94, 49.51, 47.22, 45.81, 39.49, 38.99, 38.37, 37.83, 36.92, 34.30, 31.03, 30.40, 28.71, 27.93, 26.16, 23.50, 21.76, 21.26, 20.76, 20.66, 20.60, 20.58, 20.01, 18.09, 17.61, 16.44, 16.06, 15.40.

HRMS(ESI+) m/z calcd. for C_45_H_68_O_15_[M + Na]+ 871.4456, found 871.4456.

**General procedure for the synthesis of CKN** [5, 6]**.**

The Compound 3 (50 mg, 58.9 umol)，β-Alanine ethyl ester HCl (RNH_2_) (50 mg, 0.32 mmol), NaBH(OAc)_3_ (50 mg, 0.23 mmol), and DCM (2 mL) were successively added, and the mixture was allowed to reach 0°C.,After completion of the reaction (TLC monitoring, CH_2_Cl_2_/CH3OH, 5/1, V/V, on silica gel plate), water was added, and the product was isolated by extraction with EtOAc. The organic phase was washed with water, brine, dried over anhydrous sodium sulfate, filtered, and concentrated under vacuum to obtain the desired crude products (36mg, 68%). The appropriate compounds (4-15) were obtained following purification by silica gel column chromatography to give a white powder (28 mg, 52%).

^1^H NMR (600 MHz, cdcl3) δ = 5.17 (t, J=9.5, 1H), 5.00 (t, J=9.7, 1H), 4.91 – 4.86 (m, 1H), 4.79 (td, J=10.7, 5.2, 1H), 4.67 (d, J=7.9, 1H), 4.46 (dd, J=10.9, 4.5, 1H), 4.21 (ddd, J=14.4, 7.2, 3.8, 3H), 4.17 – 4.01 (m, 3H), 3.67 – 3.57 (m, 2H), 2.78 (m, 1H), 2.45 (m 1H), 2.36 (m, 2H), 2.21 – 2.08 (m, 2H), 2.02 (ddd, J=22.5, 16.2, 7.1, 18H).

^13^C NMR (151 MHz, cdcl3) δ 170.81 , 170.59 , 170.36, 170.33, 170.29, 169.41, 168.94, 94.59, 82.92, 80.50, 75.18, 73.08, 71.77, 71.61, 68.41, 62.07, 59.60, 55.77, 52.93, 49.81, 49.51, 47.33, 45.81, 45.72, 39.49, 38.98, 38.37, 37.82, 36.92, 34.30, 31.03, 27.92, 26.16, 23.49, 21.74, 21.24, 21.04, 20.79, 20.74, 20.67, 20.64, 20.58, 20.00, 17.61, 16.43, 16.04, 15.40.

MUDI-TOF m/z calcd. for C_54_H_85_NO_18_[M + Na]+1058.5664, found 1058.4825.


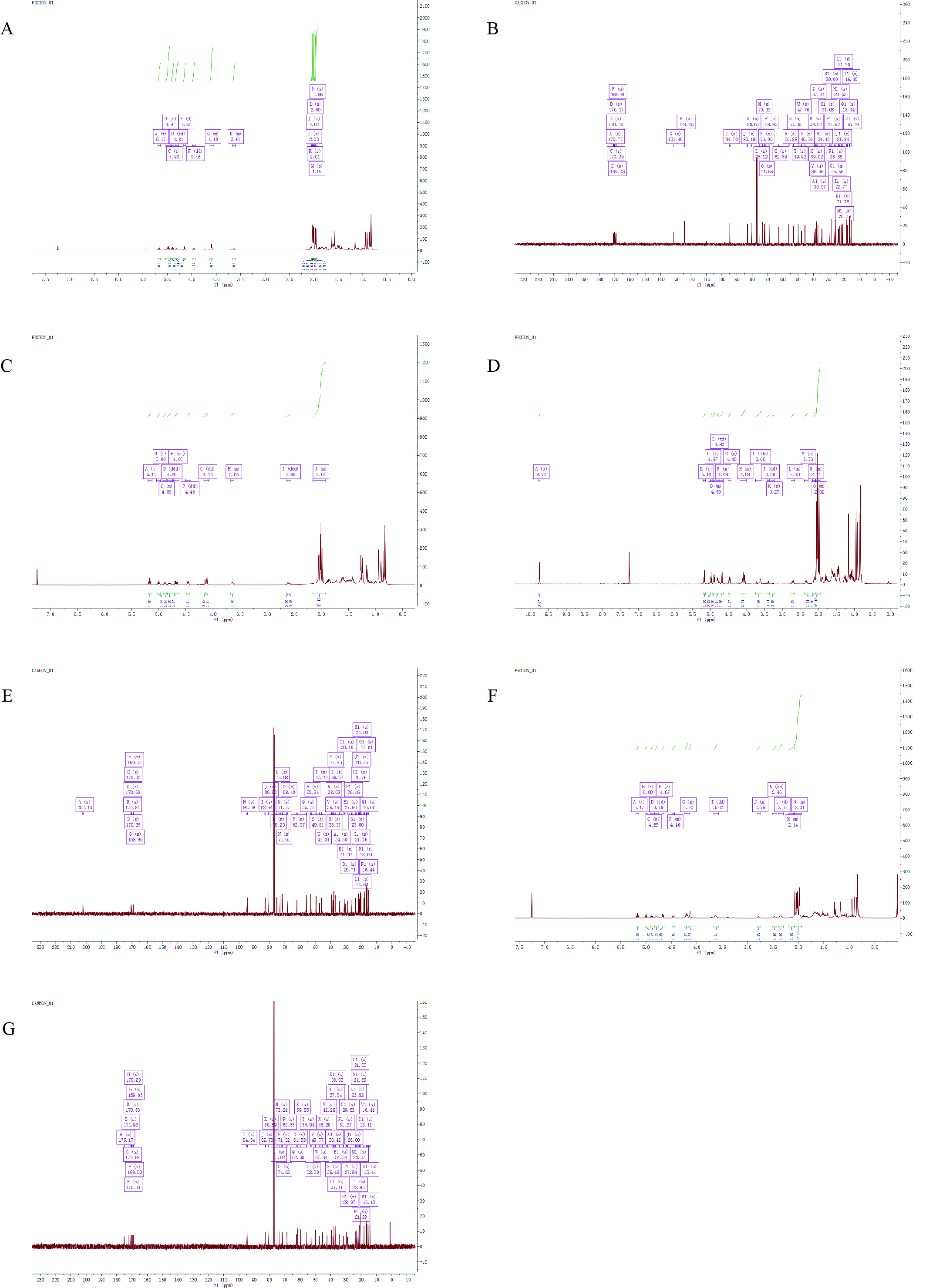


Figure S2. The NMR spectrum analysis of 1, 2, 3 and CKN. The ^1^H NMR (A) and ^13^C NMR(B) of 1, solvent: CDCl_3_. The ^1^H NMR (C) of 2, solvent: CDCl_3_. The ^1^H NMR (D) and ^13^C NMR(E) of 3, solvent: CDCl_3_. The ^1^H NMR (F) and ^13^C NMR(G) of CKN, solvent: CDCl_3_.

**Effects of different doses of CKN on atherosclerotic plaque in the aorta of high-fat fed ApoE-/- mice**

**Materials and methods**

C57BL/6 ApoE-/- mice were purchased from Peking University Health Science Center (Beijing, China). high-fat and high-choline diet were obtained from Tengxin Biotechnology Co. LTD (Chongqing, China). Oil Red O were obtained from Sigma-Aldrich (CA, USA).

**Animals and treatments**

To study the preventive and therapeutic effects of compound on atherosclerosis in vivo, Seventy-two male C57BL/6 ApoE^-/-^ mice aged 8 weeks (22–25 g) were housed under specific pathogen-free conditions on a 12-hour light-dark cycle in the animal facility at the Animal Center of the Third Military Medical University. Animals were randomly divided into five groups (n=6) and provided with unlimited access to water and high-fat and high-choline diet (basic feed containing 40% fat and 1.25% cholesterol and 0.5% sodium cholate), except the control group with basic feed. Animals were treated with Atorvastatin (3 mg/kg) CK (3 mg/kg), CKN (0.3 mg/kg, 1 mg/kg, 3 mg/kg) and by intra-peritoneal injection once a day. After 10 weeks, the mice were fasted overnight and sacrificed by CO_2_ inhalation in accordance with the AVMA guidelines for the euthanasia of animals, 2013 edition. Samples were collected for the subsequent experiments.

**Image analysis of aortic**

For en face analysis, the thoracic aorta were opened longitudinally and fixed by 10% formalin. The samples were stained with Oil-Red O for 15 minutes and washed. The sections were examined under a light microscope Images were captured with Laser Confocal Microscope (Nikon Eclipse 90i light microscope), Image-Pro Plus 6.0 software were used to aortic tissues quantitative analysis.


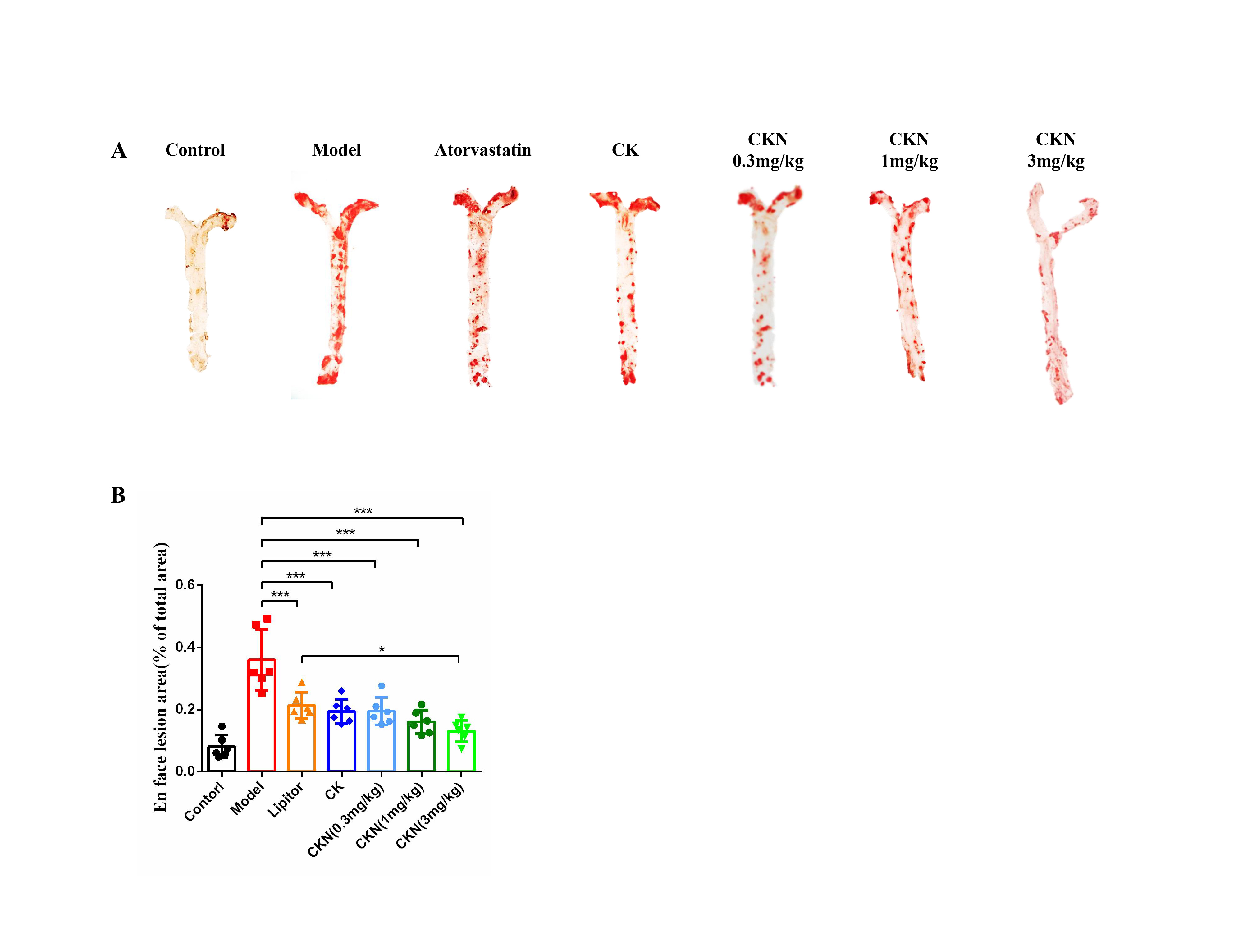


Figure S2. Effencts of different administration doses of CKN on atherosclerotic plaques in AopE^-/-^ mice. Representative images of thoracic aortic lesions (A) and quantification of atherosclerotic lesions shown as percentage of thoracic aorta (B).

**References**

[1] K. M. Mennie, S. M. Banik, E. C. Reichert, et al., Catalytic Diastereo and Enantioselective Fluoroamination of Alkenes. J Am Chem Soc. 140 (2018) 4797-802.

[2] T. H. M. Wong, X. G. Li, D. K Ma, et al., HNTf_2_-Catalyzed Synthesis of Hydrodibenzofurans by an Epoxidation/Semipinacol Rearrangement Cascade. Org Lett. 22 (2020) 1950-54.

[3] T. Chen, W. J. Liu, W. Gu, et al., Dynamic Kinetic Resolution of β-Substituted α-Diketones via Asymmetric Transfer Hydrogenation. J Am Chem Soc. 145 (2023) 585-99.

[4] [E. de. Orbe](javascript:{}), [E. Antonio](javascript:{}), Broadening the Scope of the Gold-Catalyzed [2+2] Cycloaddition Reaction: Synthesis of Vinylcyclobutenes and Further Transformations. EUR J ORG CHEM. 22 (2018) 2740-52.

[5] E. D. Mock, I. Kotsogianni, W. P. F. Driever, et al., Structure-Activity Relationship Studies of Pyrimidine-4-Carboxamides as Inhibitors of N-Acylphosphatidylethanola

-mine Phospholipase D. J Am Chem Soc. 64 (2020) 481-515.

[6] R. J. Griffiths, W. C. Kong, S. A. Richards, et al., Oxidative β-C-H sulfonylation of cyclic amines. CHEM SCI. 9 (2018) 2295-2300.
